# Complete genome sequence of human papillomavirus type 45 identified from a cervical cancer patient in Bangladesh

**DOI:** 10.1128/mra.01382-25

**Published:** 2026-03-10

**Authors:** Md. Touki Tahamid Tusar, Rifat Ara, Raduyan Farazi, Chonda Rani Roy, Roni Mia, Anandha Mozumder, Anupam Das, Safayet Hossain Piyel, M. Shaminur Rahman, Mohammad Imtiaj Uddin Bhuiyan, Sharmin Akter, S. M. Rashed Ul Islam, Md. Amimul Ehsan, Muzahed Uddin Ahmed, Md. Golzar Hossain

**Affiliations:** 1 Department of Microbiology and Hygiene, Bangladesh Agricultural University 54492, Mymensingh, Bangladesh; 2 Department of Gynecology and Obstetrics, Dhaka Medical College 468779 , Dhaka, Bangladesh; 3 Department of Microbiology, Jashore University of Science and Technology 421984, Jashore, Bangladesh; 4 Department of Environmental Health Science, University of Georgia 1355, Athens, Georgia, USA; 5 Department of Physiology, Bangladesh Agricultural University 54492, Mymensingh, Bangladesh; 6 Department of Virology, Bangladesh Medical University 846579, Dhaka, Bangladesh; 7 Department of Medicine, Bangladesh Agricultural University 54492, Mymensingh, Bangladesh; DOE Joint Genome Institute, Berkeley, California, USA

**Keywords:** human papillomavirus type 45, cervical cancer, complete genome sequence, Bangladesh

## Abstract

Cervical cancer (CC) is the second most common cancer among Bangladeshi women and caused by persistent infection with high-risk human papillomaviruses (hrHPVs). Here, we report the complete genome sequence of HPV type 45 (HPV45) identified from a CC patient in Bangladesh.

## ANNOUNCEMENT

Human papillomavirus (HPV) is the primary etiological agent of cervical cancer (CC), the second most common cancer among Bangladeshi women, accounting for 12% of all female cancers (1). HPVs are non-enveloped viruses with small, double-stranded, circular DNA genomes of approximately 8 kb containing eight open reading frames (ORFs) (2). HPVs are classified within the family *Papillomaviridae*, which includes 53 genera, of which five (*Alpha*, *Beta*, *Gamma*, *Mu*, and *Nu*) are capable of infecting humans (3). Although reports have shown that high-risk human papillomavirus (hrHPV) infections with various genotypes, such as 16, 18, 31, 45, 53, and 66, are predominant in Bangladesh (1), no complete genome sequence of HPV from CC patients in Bangladesh has previously been available.

A cervical swab was collected in phosphate-buffered saline from a 42-year-old CC patient in February 2025 at the National Institute of Cancer Research & Hospital, Dhaka, Bangladesh. DNA was extracted using the TIANamp Virus DNA/RNA Kit (Tiangen, China) following the manufacturer’s protocol. Next-generation sequencing (NGS) was carried out on the Illumina NovaSeq X Plus System. Metagenomic libraries were prepared following the iNextEra protocol, including DNA tagmentation with Illumina bead-linked transposomes, adapter ligation, and limited-cycle PCR for indexing (4). The libraries were purified using AMPure XP beads, quantified with a Qubit Flex Fluorometer, and sequenced at Novogene to produce 2 × 150 bp paired-end reads (5). A total of 27,557,952 input reads were obtained.

FastQC (v0.11.6) was used to perform quality control of the FASTQ files (6). Adapter trimming and removal of low-quality reads were performed using Trimmomatic v0.39 (7), with a sliding window of 4, a minimum average quality score of 20, and retention of reads ≥40 bp in length. To deplete host DNA, quality-filtered reads were aligned to the *Homo sapiens* reference genome (GRCh38, NCBI RefSeq accession GCF_000001405.40) using BBMap v39.35 (8). The remaining unmapped reads were aligned to the HPV45 reference genome (NC_075269.1; genome size: 7,858 bp) using BWA-MEM v0.7.17. Sorting and indexing were performed with SAMtools v1.12 (9), while the consensus genome was generated using BCFtools v1.12 and VCFtools v4.1 (10). Genome annotation was conducted by aligning the newly assembled genome to the HPV type 45 reference genome (NCBI RefSeq: NC_075269.1). All tools were run using default parameters, unless otherwise specified. The completeness of the assembled consensus genome was validated by identifying terminal repeat structures and confirming raw read support spanning the contig ends.

The sequencing data underwent quality control and host filtering. Trimmomatic retained 97.36% (13,415,201 pairs) of the 13,778,976 original read pairs. Subsequent host read removal using bbduk yielded 1,691,514 reads (6.14% of the total) for downstream analysis. A subset of these reads (8,958) mapped to the reference sequence (NCBI RefSeq: NC_075269.1), providing 166× mean depth of coverage across 99.8% of the 7,858 bp genome with GC content 39.72%.

The assembled genome shows 99.20% identity with the reference genome, as determined by BLASTn. Genome annotation identified 8 ORFs, representing the complete set of genes characteristic of an HPV45 isolate. This complete genome of HPV45 will contribute to global genomic data sets and enhance our understanding of the pathogenic and epidemiological characteristics of HPVs in Bangladesh.

## Data Availability

The complete genome sequence of HPV_MGH-TT01 has been deposited in GenBank under accession number PX473505. The BioSample, BioProject, and SRA accession numbers are SAMN52919474, PRJNA1348789, and SRR37115695. Codes are available at https://github.com/SShaminur/Viral-Variant-and-Concensus-Calling-Pipeline-from-Metagenome-virvarcall-pipeline.
